# Augmented Reality in Medical Practice: From Spine Surgery to Remote Assistance

**DOI:** 10.3389/fsurg.2021.657901

**Published:** 2021-03-30

**Authors:** Fabio Cofano, Giuseppe Di Perna, Marco Bozzaro, Alessandro Longo, Nicola Marengo, Francesco Zenga, Nicola Zullo, Matteo Cavalieri, Luca Damiani, Daniya J. Boges, Marco Agus, Diego Garbossa, Corrado Calì

**Affiliations:** ^1^Neurosurgery Unit, Department of Neuroscience “Rita Levi Montalcini,” University of Torino, Turin, Italy; ^2^Spine Surgery Unit, Humanitas Gradenigo, Turin, Italy; ^3^Spine Surgery Unit, Humanitas Cellini, Turin, Italy; ^4^Spine Surgery Unit, Casa di Cura Città di Bra, Bra, Italy; ^5^Intravides SRL, Palazzo degli Istituti Anatomici, Turin, Italy; ^6^LD Consulting, Chiavari, Italy; ^7^BESE Division, King Abdullah University of Science and Technology, Thuwal, Saudi Arabia; ^8^College of Science and Engineering, Hamad Bin Khalifa University, Doha, Qatar; ^9^Neuroscience Institute Cavalieri Ottolenghi, Orbassano, Italy; ^10^Department of Neuroscience “Rita Levi Montalcini,” University of Torino, Turin, Italy

**Keywords:** augmented reality, telementoring and surgery, spine surgery, hologram 3D display, remote assistance, COVID emergency, AR surgery, remote proctor

## Abstract

**Background:** While performing surgeries in the OR, surgeons and assistants often need to access several information regarding surgical planning and/or procedures related to the surgery itself, or the accessory equipment to perform certain operations. The accessibility of this information often relies on the physical presence of technical and medical specialists in the OR, which is increasingly difficult due to the number of limitations imposed by the COVID emergency to avoid overcrowded environments or external personnel. Here, we analyze several scenarios where we equipped OR personnel with augmented reality (AR) glasses, allowing a remote specialist to guide OR operations through voice and *ad-hoc* visuals, superimposed to the field of view of the operator wearing them.

**Methods:** This study is a preliminary case series of prospective collected data about the use of AR-assistance in spine surgery from January to July 2020. The technology has been used on a cohort of 12 patients affected by degenerative lumbar spine disease with lumbar sciatica co-morbidities. Surgeons and OR specialists were equipped with AR devices, customized with P2P videoconference commercial apps, or customized holographic apps. The devices were tested during surgeries for lumbar arthrodesis in a multicenter experience involving author's Institutions.

**Findings:** A total number of 12 lumbar arthrodesis have been performed while using the described AR technology, with application spanning from telementoring (3), teaching (2), surgical planning superimposition and interaction with the hologram using a custom application for Microsoft hololens (1). Surgeons wearing the AR goggles reported a positive feedback as for the ergonomy, wearability and comfort during the procedure; being able to visualize a 3D reconstruction during surgery was perceived as a straightforward benefit, allowing to speed-up procedures, thus limiting post-operational complications. The possibility of remotely interacting with a specialist on the glasses was a potent added value during COVID emergency, due to limited access of non-resident personnel in the OR.

**Interpretation:** By allowing surgeons to overlay digital medical content on actual surroundings, augmented reality surgery can be exploited easily in multiple scenarios by adapting commercially available or custom-made apps to several use cases. The possibility to observe directly the operatory theater through the eyes of the surgeon might be a game-changer, giving the chance to unexperienced surgeons to be virtually at the site of the operation, or allowing a remote experienced operator to guide wisely the unexperienced surgeon during a procedure.

## Introduction

The challenges of learning, planning and performing procedures in spine surgery have been enriched by the recent development of new technological tools and instrumentations, able to assist surgeons and reducing surgical invasiveness (Minimally-Invasive Surgery, MIS) but maintaining a valuable profile of safety (1–5). One of the most promising applications of advancements in visual/haptic display technologies and computational power is represented by augmented reality (AR) (6), an emerging technological field. After the developments and further drops of prices for the Virtual Reality (VR) headsets, few companies have started the development of AR glasses. First commercial AR headsets available on market were the Epson Moverio BT-200, allowing imaging superimposition thanks to an integrated camera and tracking systems. The advantage of this system, now updated and evolved with better sensors, is to be able to interface with Unity, a game engine that can be used to create custom tools for 3D visualization and tracking and that became popular thanks to VR and gaming industry. Also, the possibility of stereoscopic vision allows projection of three-dimensional objects on the user eyesight; superimposition of digital content to the real field of view creates a digital hologram, which can be informative of the observed reality.

While performing surgeries in the operating room (OR), surgeons and assistants often need to access several information regarding surgical planning and/or procedures related to the surgery itself, or the accessory equipment to perform a wide spectrum of operations (7). Furthermore, as known, the shape and timing of surgical learning curve for surgeons strictly relies on the possibility to physically access the OR and learn procedures from other experienced colleagues in a space/time-dependent and limited manner; all these processes could be eased by AR.

Interactions with such digital objects were something considered science fiction, as seen in movies projecting us in the future; nevertheless, recently Microsoft implemented this technology with the “hololens,” an AR visor with a tracking system able to recognize hand motion thus allowing interactions with holograms. Most likely this technology will access the general consumer market within the next 10 years. Our group has already large experience with mixed reality, having worked on one of the first large-scale setups for AR interactions “CAVE” (8), which was at the basis of the idea of engineering a portable system projecting hologram to assist neurosurgery.

Generating 3D models from medical images does not imply similar challenges compared to electron micrographs (8–12) (segmentation of the latter type requires knowledge from the user of the observed image, and generation of masks could take longer, although semi-automated or fully automated techniques can speed up the process) (12). On the contrary, medical images such as CT scans or MRI are often black and white images, that could be easily binarized and hence used to generate directly a three-dimensional object. Here, we propose to use techniques used for segmentation of microscopy images to clinical medical images, in order to generate 3D dimensional models that could be used as holograms to be projected on stereoscopic AR glasses, allowing the visualization of models with integrated surgical planning.

Another practical case for the use of the AR was to face the number of limitations imposed by the COVID emergency. Indeed, during months of hard lockdown, until recently, access to OR was limited, with strict regulations regarding personnel allowed to enter surgical theater. For several procedures, external experts or consultants were needed to assist for specific procedure, like setting up special equipment, or assist during surgery for the implant of new devices. Since access to the OR was not free to specialists, AR came in handy by allowing these experts to pilot these particular operations directly.

In this paper several scenarios of AR-assisted spinal procedures are presented, in order to show and describe all the potential benefits and caveats in the processes of mentoring, coaching and assistance to the surgical staff. We were able to demonstrate how AR is beneficial during special surgical procedures. The flexibility and easiness to use of the software platform makes the system suitable for multiple devices; AR have the potential to make this setup a standard equipment in the OR, such as surgical scissors and scalpels.

## Methods

This study is a preliminary case series of prospective collected data about the use of AR-assistance in spine surgery from January to July 2020. The technology has been used during surgeries for lumbar arthrodesis in a multicenter experience involving author's Institutions.

## Cohort

We selected a cohort of 12 patients that required lumbar arthrodesis surgery for degenerative lumbar spine disease.

## Imaging and 3D Reconstruction

CT Scans used to classify and plan surgery were acquired carefully using a z spacing allowing smooth 3D reconstructions without visible artifacts during the renderings. Image segmentations and 3D reconstructions were obtained either using a pipeline developed for electron microscopy stacks at nanometer resolution (11, 13) or with the Horos software, available for free.

## Augmented Reality Headsets

**Figure 1:**
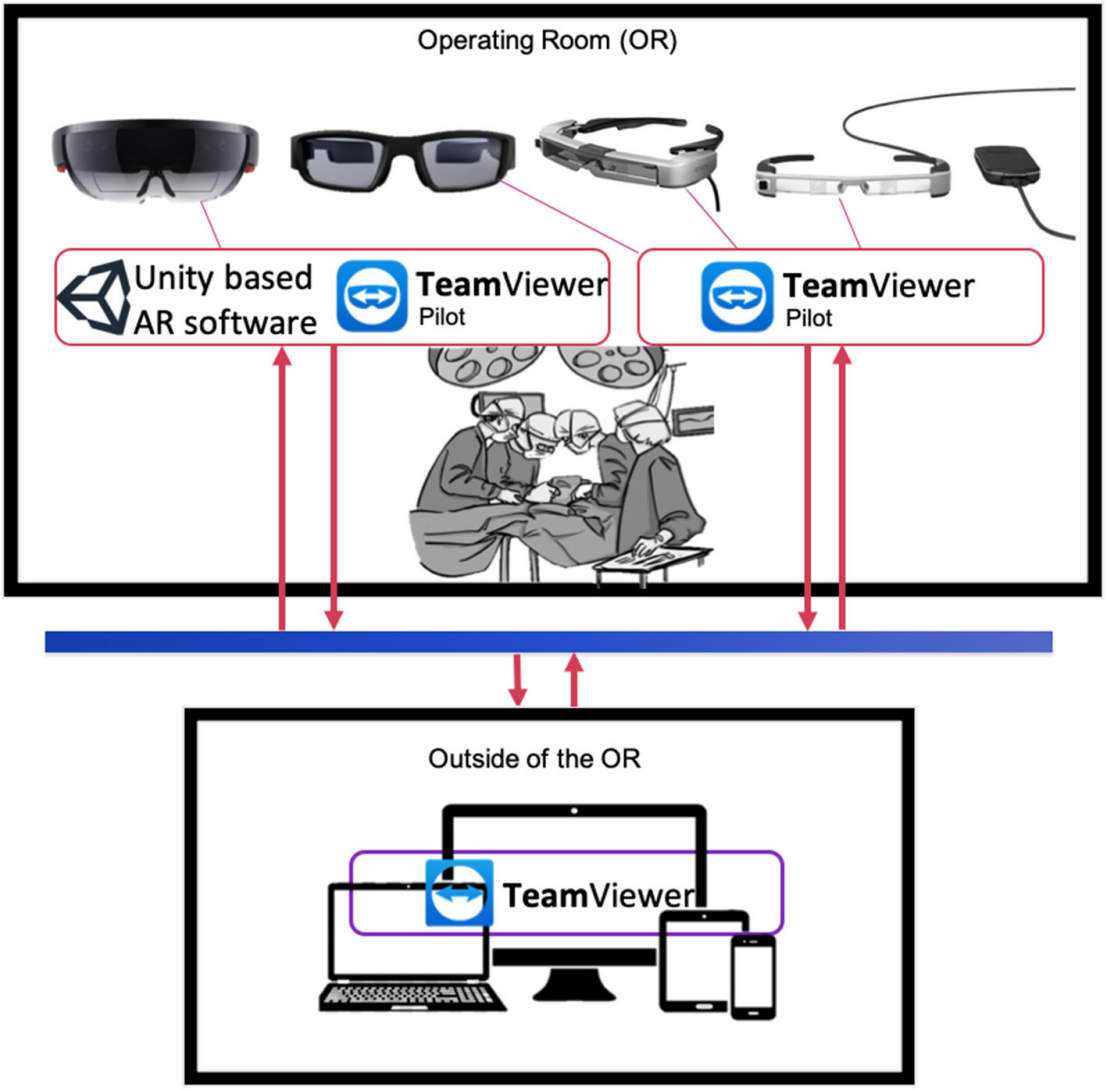
Graphical representation of AR information flow between OR and remote users. Top panel: key personnel in the OR (i.e., physician, technical specialists, surgeons) wearing AR goggles equipped with software for digital content superimposition (Unity custom tool) and/or video streaming and interaction (e.g., Teamviewer Pilot) from the OR. Goggles models from the left: Microsoft Hololens, Vuzix Blade, Epson BT-350, Epson BT-300. Bottom panel: personnel outside the OR can visualize the video streaming from goggles equipped with Teamviewer Pilot via TeamViewer app on laptops, tablets or smartphones.

In order to visualize digital content, or participate to an interactive session using augmented reality (AR), we took advantage of four different state of the art AR goggles: the Epson BT-300 and BT-350, both allowing HD projection, with a 5 MPx camera on board, and the Vuzix Blade, also allowing HD projection (Figure 1). The latter is equipped though with a 8 Mpx camera on board, allowing higher resolution video streaming, which is then better suited to visualize surgical details provided by the first operator. All these systems are wearable with ease, and can be used with TeamPilot app, allowing to send the audio-video stream to a remote user running the TeamViewer app on a pc, smartphone or tablet (Figure 1). Remote users can take snapshots and create visual clues such as arrows or doodles on a still frame that can be visualized on the eye of the user wearing the goggles (Supplementary Video 1). Despite the use of different headsets, powered by different head-mounted display (HMD) technology, all of them were running the same software tool (see next section Software Tools). This allowed us to assess the use of the technique, rather than the headset technology *per se*. For one case we have used Microsoft Hololens 1, which are equipped with a 8MPx camera and HD stereoscopic projection. To take full advantage of the stereoscopic view of the system, we developed a custom-made app using Unity.

## Software Tools

TeamViewer pilot is a cross platform remote assistance software that was developed and enhanced for the purpose of exploiting AR and AI features combined (Figure 1). Key personnel wearing the AR goggles operate with TeamViewer Pilot at one end, interacting with other users equipped with a TeamViewer remote client at another one, running on a laptop or tablet. Both software applications require fast connections in order to perform relatively smooth. As part of the routinely preparations of the OR, it is recommended to check on any updates that may occur to the OR access link to its Internet Service Provider (ISP) to limit the occurrence of technical issues during the operation. A dedicated connection link is also desired. Our setup has tested an average bandwidth and delay values of 60 Mbps downstream, 90 Mbps upstream and a ping value of 50 ms. Other parameters that need to be adjusted within the TeamViewer software involves hardware acceleration options in the case of systems with weak GPUs. TeamViewer will automatically attempt to optimize its performance based on balancing between connection and image quality. This can be solely controlled by the enduser as well.

Another in-house Unity-based software tool customized specifically for the HoloLens goggles is the Holosurgery app. This piece of software holds features that enable more convenient input methods such as hand gestures and voice commands. Summoning optimized pre-processed imaging data such as 3D models of a patient's spine is achieved with simple key vocal inputs, e.g., “Show 3D Model.” In addition, the 3D model is manipulated using hands and fingers motions and that achieves re-scaling, movement, and rotating of the 3D model. There is also more complex geometry operations such arbitrarily clipping planes which neatly visualizes a clipped region of interest within the displayed 3D model.

All three state of the art software tools along with the implemented AR goggles empowers the OR staff to perform normally in non-normal and challenging times similarly in the case of global pandemics.

## System Usability Scale

System Usability Scale is an industry standard used to give a gross but reliable evaluation of the usability of a product. It is a questionnaire that can be customized to a certain extent, based on individual needs. Each answer requires an answer on a scale from 1 (strongly disagree) to 5 (strongly agree). A 9 questions questionnaire reported in Table 1 was administered to *n* = 5 expert who have used to devices in the OR.

**Table 1:** System usability scale (SUS).

**System usability scale (SUS)**	***N* (1–5)**
I think I would like to use the Augmented Reality (AR) system frequently	
I found the AR application unnecessarily complex	
I think that I would need technical support for using AR goggles	
I like using the AR interface	
I think that most people would learn to use this system quickly	
I felt very confident using the AR system	
I needed to train a lot before I could use the AR system	
The information provided by the interfact was clear and helpful	
I felt is difficult to interact and control the system	

## Case Studies

### Telementoring

**Figure 2:**
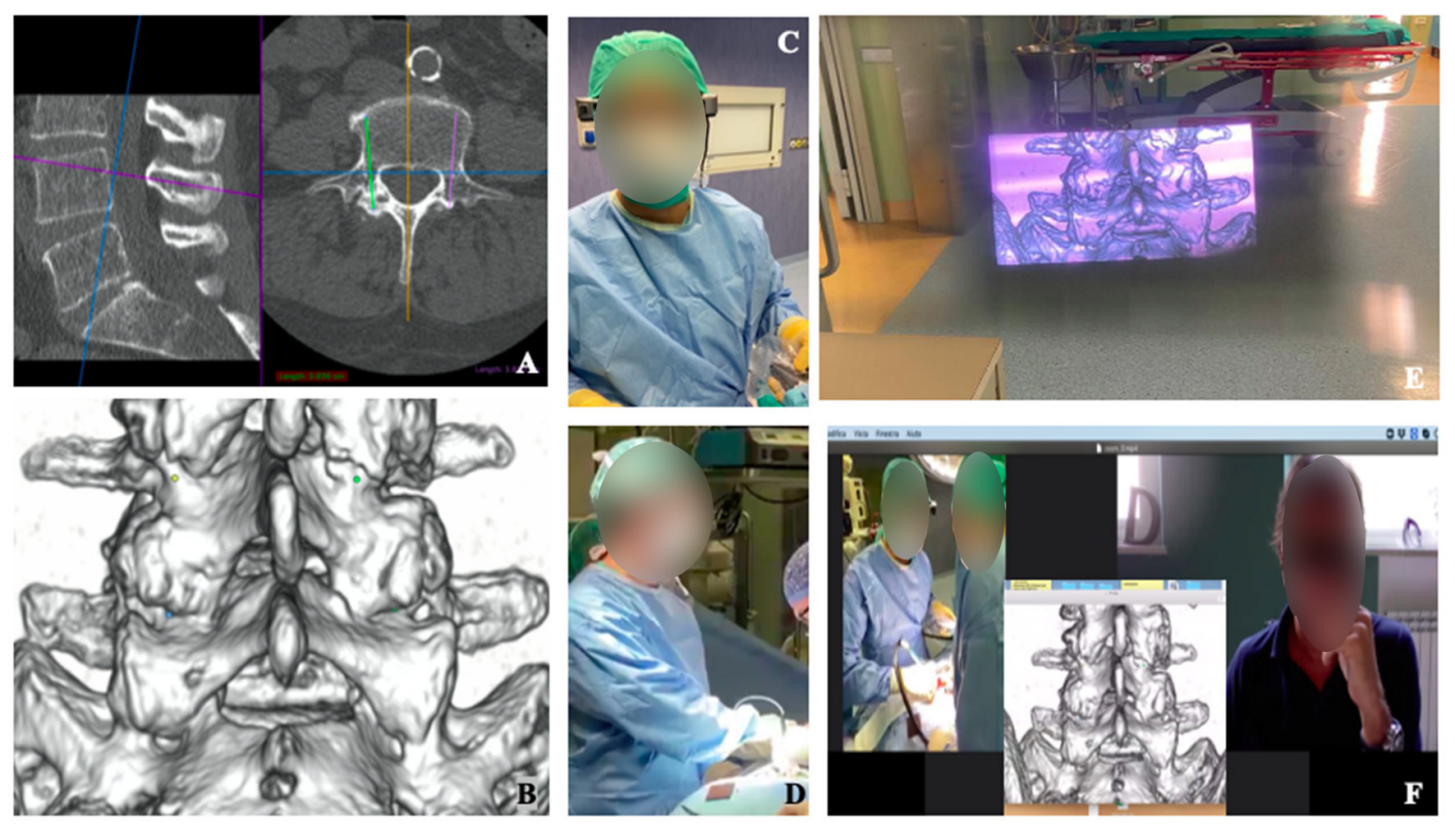
Visualization of intra-operative 3D-model planning **(A–D)**. Surgical planning of screws positioning for lumbar spine fusion is shown **(A)**, with 3D reconstructed model highlighting the screws' entry points **(B)**. Surgeon wore smart glass during surgery **(C,D)** and, with augmented reality, was able to see the 3D model wherever He preferred into the space **(E)**. The enhanced videoconference function with smart glasses' screen sharing allowed participants to see through the eyes of the surgeon and communicate with him **(F)**.

**Figure 3:**
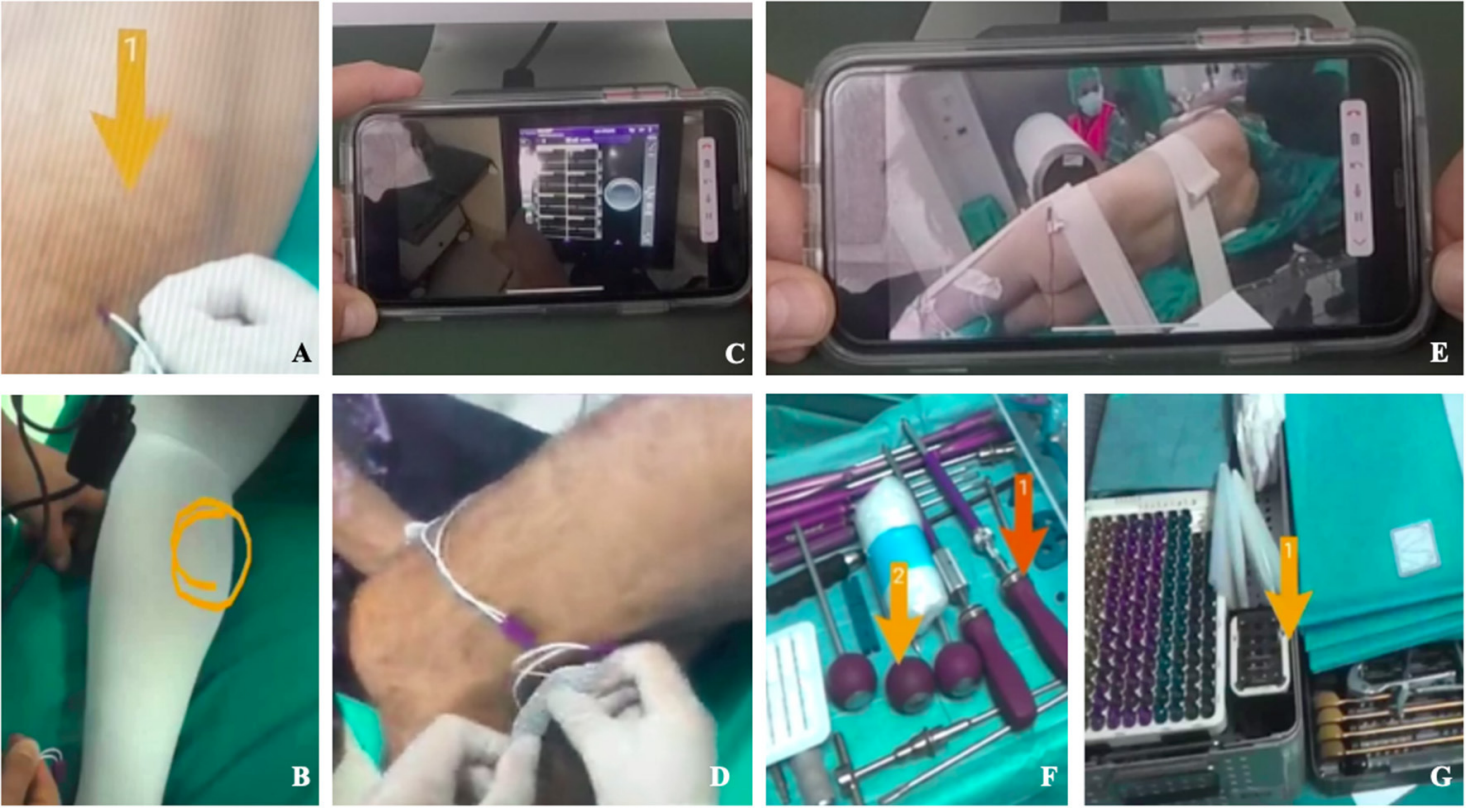
Remote operative Room setup with Epson smart glasses **(A–G)**. Remote vision of the operative room showing neuromonitoring electrodes positioning **(A,B,D)** and enhanced videoconference function that allowed to avoid the physical presence of specialists consultants in the OR **(A–D)**. Remote vision of patient positioning and instrumentation setting in the OR using enhanced videoconference function **(E–G)**.

AR goggles allowed to stream videos and transmit still images from the surgical field to different specialists (Figure 2). Processes of supervision and coaching have been performed to verify the possibility of an effective and interactive remote-assistance in the OR without requiring a physical presence. The use of AR googles goes beyond simple video-conferencing, since remote users can interact with the video stream and make drawings or create arrows that the user wearing the device can visualize live. This gives the possibility to not only give audio, but also visual clues to the operator in the OR. AR goggles have been used also to face physical limitations during the COVID emergency to allow OR technicians and technical consultants from spinal devices companies supervise—before and during the procedures—surgeons, nurses, and neurophysiologists without accessing the OR (Figure 3). Spinal instrumented procedures require specific surgical instruments, both for the positioning of implants (e.g., screws, rods, or cages) and to allow surgeons to approach the spinal canal and/or during the decompressive step (Figure 3). Neuronavigation could be used to improve the accuracy of screw positioning if compared with the free-hand technique (14). Furthermore, intraoperative neuromonitoring during spinal procedures has become one of the most important tools to preserve the integrity of the nervous structures, especially for MIS techniques (15).

### Surgical Planning

Surgeons had the possibility to get a live visualization of the CT reconstruction and of the planned trajectories (Figure 2) (7) of the screws while maintaining the view on the surgical field. Moreover, Microsoft hololens allow interactions with gesture by hand tracking, which allows to keep the surgical theater sterile.

### Teaching

The ability to obtain an ergonomic live-sharing of surgeons view, together with the possibility to overlay images or videos offered the opportunity to involve a group of young residents and medical students for a remote step-by-step interactive learning of the surgical procedure (Figures 2, 3).

## Results

A total number of 12 lumbar arthrodesis have been performed while using the described AR technology. Five cases of Lateral Lumbar Interbody Fusion (LLIF) and 7 Transforaminal Lumbar Interbody Fusion (TLIF) were performed with posteriore screwing through Standard (5 pts, PT) or Cortical Bone Trajectory (2 pts, CBT). Intraoperative neuromonitoring was used in all the cases. Neuronavigation was used in two TLIF procedures.

### Telementoring

In three cases (2 CBT-TLIF, 1 LLIF) surgical procedures have been shared through enhanced videoconferences among three experienced surgeons. The surgeon in the OR discussed the case while showing the screw entry-point and the trajectory, with the aid of the fluoroscopy and neuromonitoring. In the LLIF case, the discussion about the procedure involved the lateral positioning, the trans-psoas approach and the cage placement. In seven cases (5 LLIF and 2 TLIF procedures) the positioning of neuromonitoring electrodes on patients skin and the wires connection to the central monitoring platform and display was made by surgeons wearing AR goggles with the remote assistance from specialized technicians. Similarly, the remote assistance allowed the surgeon to set neuronavigation in two cases. In all the procedures, companies ensured a live support for nurses assisting surgeons with regard to the devices and surgical instrumentations needed.

### Surgical Planning

In three cases AR goggles allowed the surgeon to access to the surgical planning of patients that underwent CBT fixation in real time while maintaining the view on the operator field.

### Teaching

In two cases (1 LLIF, 1 TLIF) a group of four residents belonging to their first year of the Residency program and two medical students got access to the procedure with a remote connection, with the possibility to interact with the surgeons. Surgeries were performed in a step-by-step manner.

**Figure 4:**
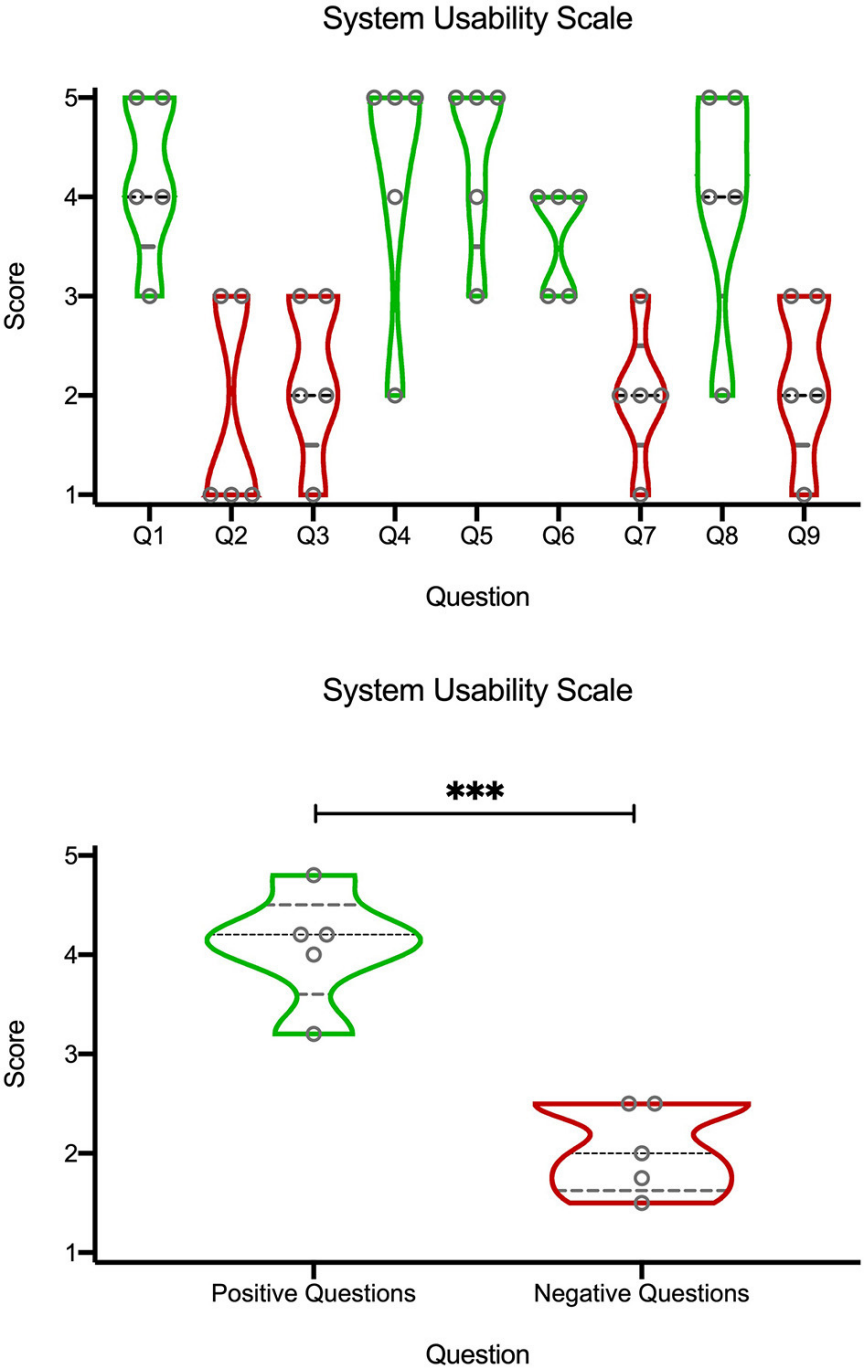
Violin plots of quantification of the SUS questionnaire (Table 1) on a Likert scale (1 corresponding to “strongly disagree,” and 5 to “strongly agree”). Black dashed line represents the median, the gray dashed lines represents the quartiles, and width of the violin corresponds to the number of points at a certain height. The top graph are the scores from individual questions. Bottom graph is an average from “positive” (green) or “negative” (red) questions. ***p < 0.01, unpaired t-test.

No complications potentially linked to the use of AR were registered, such as malfunction of the neuromonitoring and of the neuronavigation system, or infections. Surgeons reported a positive feedback as for the ergonomy, wearability and comfort during the procedure, as confirmed by the results shown on the graphs in Figure 4 after SUS questionnaires.

## Interpretation

AR represents the possibility to create a useful and real-time interaction between multiple environments and/or images/videos of interest (16). AR systems have been conceived and developed during the last decades and their applications for medicine have been described for different specialties such as neurosurgery, radiotherapy, orthopedics or plastic surgery. First examples of application and implementation of AR in neurosurgery were described by Roberts et al. in 1986 which proposed the projection of CT images in a surgical microscope. In 1998 the same principles were used to project vascular structures with fluoroscopy while in 2002 AR was applied in a neurosurgical endoscope (17).

In this case series a simple, ergonomic and successful use of AR goggles is presented. In addition, the unfortunate conjunction with the COVID-19 pandemic has led to the chance of facing physical restrictions adding further applications of this technology.

These tools, indeed, allow surgeons to view images and use apps anywhere and anytime they like overlaying digital content on their real field of view. Moreover, images, videos or screens shared by other devices could be watched on these see-through lenses, through an enhanced videoconference app (Supplementary Video 1). Considering this last feature, different preliminary applications of EPSON ECC in surgery have already been described and the idea of telementoring with augmented reality took place. Recently, Roja-Munoz et al. published their experience with the STAR (System for Telementoring with Augmented Reality) system, analyzing different results of two different groups that performed leg fasciotomies. Participants were unexperienced surgeons (surgical residents and medical students) and were divided into two different groups: the former receiving remote instructions provided by an expert surgeon, directly on their field of view, using the STAR system; the latter receiving no external guidance beyond initial consultation of the Advanced Surgical Skills for Exposure in Trauma course manual. Results showed fewer mistakes and better performances among mentees belonging to the group that received guidance trough the STAR system (18).

Another important advantage offered by this AR system, is the real-time visual feedback of the operative field that allows the mentor to provide a better coaching, as reported in other previous papers (19, 20). Davis et al. described an interesting experience using the Virtual Interactive Presence and Augmented Reality (VIPAR) system that allows a remote surgeon to communicate visual and verbal information in real time to a local surgeon performing a procedure; namely neurosurgeons based in Birmingham, Alabama, successfully assisted neurosurgeons in Ho Chi Minh City, Vietnam, in fifteen cases of endoscopic third ventriculostomy with choroid plexus coagulation. Neurosurgeons using the system reported a good feedback and concluded it was useful for safer procedures compared to standard operations (21). In this experience, the use of AR goggles allowed remote surgeons to follow and discuss the procedures in their crucial steps, during the approach and the device positioning phase.

These examples could represent a starting point to better investigate the potential development of AR for the teaching/supervision of surgical techniques, reducing the need for physical presence of experienced surgeon and consequently its related constraints on time and budgets.

The remote mentoring could also be considered to coordinate the setting up of the operative room for newer procedures or to help surgeons with the use of new instrumentations, even when specialist consultants could not physically enter the operative room. It is well-known that many traditional neurosurgical procedures often required the use of intraoperative neuromonitoring (IONM) in order to guarantee the best result, both in terms of extent of resection and neurological safeguarding. With the advent of new emerging minimal invasive techniques for spinal degenerative disease (e.g., CBT or LLIF), this need has spread further. Consequently, the great spread of the use of these techniques has increased the need for IONM (15, 22), with an augmented request for specialist consultants and technicians helping surgeons during the operative room set up. Thus, the other advantages of using an AR device described in this series was represented by the remote interaction between specialist consultants, surgeons and nurses, allowing the right setting of the operative room, even when advanced instrumentations are used.

Finally, and as already mentioned, although the remote mentoring and specialist counseling with the AR seemed, until few months ago, only a window on the future of the operative rooms and surgical activities, the recent dramatic experience of lockdown due to COVID-19 pandemic spread has changed the perspective, making it an everyday tool for the OR.

Another important application of AR for surgery is represented by its role in surgical planning. In this series CBT planning was visualized by the surgeon while maintaining the view on the surgical field and obtaining a real time feedback of the planned screw entry points. During the past few decades, several tools have been developed to improve pre-operative surgical planning both for spine and cranial surgery (7, 23).

The 3D printing era brought most surgical fields to an advanced new level, where even minimal differences from standard anatomy are detected, helping surgeons during the pre-operative planning and during the procedure, and then, leading to a customized surgical management. Nowadays, the so-called image guidance surgery is widely used in different surgical specialties (e.g., plastic free flap surgery, colon-rectal surgery) but recently, due to reached high accuracy, have been widely implemented in neurosurgery for cranial, spinal and skull base procedures (24). Penner et al. described their experiences with 3D model for surgical planning of cortical bone trajectory (CBT) screws positioning (7). Creating a customized spine CT scan-based 3D model, indeed, significantly improved the accuracy of screws positioning with the free hands technique, compared with the standard technique (7). The proposed methodology shares various similarities with virtual reality systems for surgical simulation, popularized in last two decades, and nowadays routinely employed for training specific interventions involving specific skills and eye-hand coordination (25).

To this end, systems incorporating haptic feedback for realistic rendering of contact forces experienced during the interaction with tissues are considered of fundamental importance for speeding up the learning curve (26). On the other side, according to the surgical specialty considered, these systems can make trainees deal with various complex hazards, rarely occurring in practice in OR, but potentially very dangerous if not carefully faced. This is especially the case of specialties involving drilling or burring, like mastoidectomy (27), orthognathic (28) and dental implantation (29), and orthopedic surgery (30).

For these tasks, haptic rendering is required to provide realistic forces and torques created by the complex interactions between the surgical tools and tissues involving tool penetration, tissue removal, rotational speed and vibrations (31). The accurate simulation of these interactions is technically challenging, since the frequency requirement for providing an adequate real time haptic feedback is above 500 Hz, corresponding to the generation of a force/torque sample every 2 ms, and the haptic simulation needs to be synchronized with visual rendering and other physical simulations eventually involving fluids and soft tissues (32).

Apart of these considerations, the proposed system can be used for gathering data related to surgical tool trajectories that can be used for fitting haptic models describing the tool-tissue dynamics that can be derived through contact models (33) or more modern machine learning methods (34). This represents a challenging and interesting research avenue that we plan to explore in the future.

Masciatelli et al. and Cabrilo et al. firstly described the application of AR in neurovascular surgery showing optimized workflow by providing essential anatomical information (35, 36). In another study by Cabrilo et al., virtual segmentations of the patient's vessels, the aneurysms, the aneurysms necks, were injected into the eyepiece of the operating microscope (37). The EPSON smart glasses could represent an innovative tool in order to integrate the production of pre-operative 3D model with the augmented reality. Once prepared, indeed, the virtual 3D model object could be loaded on the smart glasses; then, it could be scaled and positioned everywhere inside the surgeon's field of view. This way, the need to looking away from the operative field could be reduced and the neurosurgeon could be facilitated by the immediate availability of the patient's 3D model.

Resident training in surgical specialties is based on the apprenticeship model developed by Dr. William Halsted in 1980s and the training paradigm of “see one, do one, teach one” have been the pivotal concept until nowadays (38). Different authors have underlined the growing importance of introducing simulation into residents' formations and skills assessment (39). According to this picture, integrating AR into resident education could represent a renovation of the aforementioned educational model (16).

The operating theater has been the main classroom for many surgeons and is well-known that acquisition of surgical skills requires repeated occasions for hands-on practice. However, the limited number of people that can access to the OR and the large number of residents that need to learn surgical procedures often represent an issue, especially for small surgical centers and less developed countries.

Thus, the application of new technologies to increase residents' exposure to surgical procedures could play a key role for the learning curve. Thanks to its integrated camera and the previous described videoconference function, the EPSON glasses gives to the surgeon the possibility to record all the procedure and to create a live streaming that could be shared with residents and medical students, reducing the need for physical presence in the operative room. Moreover, the possibility to watch the pre-operative planning and reconstructed 3D models superimposed on the surgical field through the EPSON glasses, provide a double advantage; on one hand, indeed, surgeon has the possibility to see the model without taking eyes off of the operative field, while on the other hand, the simultaneous view of the real surgical field and of the 3D model could improve and speed up the residents' learning process.

Henssen et al. reported interesting results with their experience with AR comparing two different methods to study neuroanatomy; the classic method of studying cross sections of the brain and the one based on an AR-based neuroanatomy learning app (40). Hence, AR could represent a great instrument to improve education, especially in that fields of surgery that are particularly challenging. In neurosurgery, for example, surgeons constantly have to face with small anatomical corridors and critical neural and vascular structures that often lie within millimeters of their surgical instruments.

Understanding the true usability of the system, in order to assess whether it is not merely a technical exercise but rather a potential “everyday use tool” was key to us. In order to quantify how specialists perceived the use of the devices in the OR, they filled a SUS questionnaire (Table 1) and rated each question from 1 to 5, using a Likert scale where 1 correspond to “strongly disagree,” and 5 to “strongly agree” (Figure 4). SUS questionnaires are commonly used to rate usability of hardware or software setups (41), and their use to rate mixed reality applications is common (10). From the top violin plots we noticed a bimodal trend, around the values 4 and 2, by looking at the scores from individual questions (Top graph). Since the bimodal trend seemed to correspond to questions with a rather “positive” or “negative” meaning, we visually divided them into green (positive) and red (negative). Indeed, the positive questions (Bottom graph), related to a likeness and appreciation of the application and the devices, had higher score (around the “agree” side of the graph), while the negative questions, related to a general dislike, discomfort or unease in using the system had a general lower score (“disagree”). This semi-quantitative assessment indicated a propension of the physicians in willing to use the system as it is.

Therefore, providing a precise and reliable 3D virtual and interactive environment, AR may become an extremely valuable tool for education of neurosurgical procedures, due to their intricate and complex nature.

## Data Availability Statement

The data analyzed in this study is subject to the following licenses/restrictions: Ethical. Requests to access these datasets should be directed to Fabio Cofano, fabio.cofano@gmail.com.

## Ethics Statement

Ethical review and approval was not required for the study on human participants in accordance with the local legislation and institutional requirements. The patients/participants provided their written informed consent to participate in this study.

## Author Contributions

CC wrote the first proof of the paper, with inputs from LD and MC. FC retrieved all the relevant literature, put together all qualitative/quantitative contributions from all authors and other users, and wrote the whole manuscript. MA contributed with a critical review of the manuscript and significant inputs during the rebuttal phase. GD, AL, MB, NM, FZ, and NZ tested the AR setup on field and gave their feedbacks to the technical team. DB was responsible for the technical support of the system and coordinated the work on the Unity code for the Microsoft hololens setup, together with MC. LD and DG coordinated the operations in OR with the AR setup. DG and CC coordinated the study. All authors contributed to the article and approved the submitted version.

## Conflict of Interest

LD is the owner of the company LD Consulting. The remaining authors declare that the research was conducted in the absence of any commercial or financial relationships that could be construed as a potential conflict of interest.
